# Roles of Physiological Responses and Anthropometric Factors on the Gravitational Force Tolerance for Occupational Hypergravity Exposure

**DOI:** 10.3390/ijerph17218061

**Published:** 2020-11-02

**Authors:** Min-Yu Tu, Hsin Chu, Hsin-Hui Chen, Kwo-Tsao Chiang, Je-Ming Hu, Fang-Ling Li, Chen-Shu Yang, Chao-Chien Cheng, Chung-Yu Lai

**Affiliations:** 1 Aviation Physiology Research Laboratory, Kaohsiung Armed Forces General Hospital Gangshan Branch, Kaohsiung City 820, Taiwan; du0807@yahoo.com.tw (M.-Y.T.); charco66@gmail.com (K.-T.C.); cs5546570@yahoo.com.tw (C.-S.Y.); superbe28@gmail.com (C.-C.C.); 2 Department of Health Business Administration, Meiho University, Pingtung County 912, Taiwan; 3 Department of Life Sciences and PhD Program in Translational Medicine, National Chung Hsing University, Taichung City 402, Taiwan; 4 Institute of Medical Science and Technology, National Sun Yat-sen University, Kaohsiung City 804, Taiwan; 5 Civil Aviation Medical Center, Taipei City 105, Taiwan; hrchu@mail.ndmctsgh.edu.tw; 6 Graduate Institute of Aerospace and Undersea Medicine, National Defense Medical Center, Taipei City 114, Taiwan; 7 Department of General Medicine, Tri-Service General Hospital, National Defense Medical Center, Taipei City 114, Taiwan; orchid1319@gmail.com; 8 Division of Colorectal Surgery, Department of Surgery, Tri-Service General Hospital, National Defense Medical Center, Taipei City 114, Taiwan; jeminghu@gmail.com; 9 Graduate Institute of Medical Sciences, National Defense Medical Center, Taipei City 114, Taiwan; 10 Department of Psychiatry, Tri-Service General Hospital Beitou Branch, National Defense Medical Center, Taipei City 114, Taiwan; fanglinlee@gmail.com

**Keywords:** G force, hypergravity, heart rate, baroreflex, G-induced loss of consciousness, anti-G straining maneuver, human centrifuge

## Abstract

Gravity in the head-to-toe direction, known as +Gz (G force), forces blood to pool in the lower body. Fighter pilots experience decreases in blood pressure when exposed to hypergravity in flight. Human centrifuge has been used to examine the G tolerance and anti-G straining maneuver (AGSM) techniques of military pilots. Some factors that may affect G tolerance have been reported but are still debated. The aim of this study was to investigate the physiological responses and anthropometric factors correlated with G tolerance. We retrospectively reviewed the training records of student pilots who underwent high G training. Variables were collected to examine their correlations with the outcome of 7.5G sustained for 15 s (7.5G profile). There were 873 trainees who underwent 7.5G profile training, 44 trainees (5.04%) could not sustain the test for 15 s. The group with a small heart rate (HR) increase (less than 10%) during the first 1–5 s of the 7.5G profile had a nearly ten-fold higher failing chance compared with the large HR increase group (adjusted odds ratio: 9.91; 95% confidence interval: 4.11–23.88). The chances of failure were inversely related to the HR increase percentage (*p* for trend <0.001). Factors, including body mass index, relaxed and straining G tolerance, and AGSM, were found to be negatively correlated with the outcome.

## 1. Introduction

Modern high-performance aircraft are capable of agile maneuvers to meet the demand of acrobatic battles in the air [1]. However, military pilots are occupationally exposed to extremely high gravity environments [2,3]. Among the six axes of gravity in flight, the direction from head to toe (+Gz, commonly called G force) is the main safety concern of experts in aerospace and occupational medicine. Because hypergravity forces the blood of the pilot to pool in the lower body, the arterial blood pressure is directly affected by G force [4,5]. If the magnitude of the G force surpasses the tolerance of the human body, pilots experience stagnant hypoxia and even suffer from G-induced loss of consciousness (GLOC). While incapacitated, pilots have no control over their plane, and the consequences of GLOC could be tragic [6].

Several well-known countermeasures have been used to mitigate the risk of GLOC, including the baroreceptor reflex, anti-G suits, and anti-G straining maneuvers (AGSMs) [7,8,9]. When blood pressure at the carotid sinus declines due to acceleration, an increase in the heart rate (HR), which is regulated by the autonomic nervous system, is one of the initial compensatory physiological responses [8,10]. Forster et al. reported that changes in the HR were dependent on the level of G stress [11]. HR acceleration, primarily representing the activity of the baroreceptor reflex during rapid onset, is expected to be related to the G tolerance of the human body [10,12]. In general, the activation of HR induced by sympathetic tone takes several seconds after exposure to the G environment.

Human centrifuge training is commonly accepted as an effective and safe method to test the G tolerance of fighter pilots on the ground. Due to GLOC-related accidents, the United States Air Force (USAF) initiated a high G training program for jet pilots in 1985 [13]. The USAF prioritized pilots with conditions such as low seated systolic blood pressure (SBP), a tall and slender body, less total flying time, etc. to undergo a centrifuge ride [14]. Some former studies have demonstrated that G tolerance in the human centrifuge is positively associated with the following parameters: age, weight, flight hours, straining G tolerance, and AGSM proficiency [15,16,17]. However, the results regarding correlations between physiological responses and anthropometric factors and pilot G tolerance have been inconclusive.

To the best of our knowledge, there are few large-scale data analyses from centrifuge training investigating the correlation between trainees’ physiological responses (e.g., HR changes) and anthropometric parameters and G tolerance. To bridge this gap, we designed this study to evaluate the correlation of the physiological responses and anthropometric factors with G tolerance by analyzing a high G training database.

## 2. Materials and Methods

### 2.1. Design and Resources

We conducted this retrospective longitudinal study to investigate the associations between the G tolerance in the centrifuge, and physiological and anthropometric parameters. In Taiwan, the Aviation Physiology Research Laboratory (APRL), established in 1959, is the only professional unit responsible for implementing aviation physiology training for military aircrew. In 1996, due to the introduction of modern fighters, APRL began to provide high G training for all fighter pilots.

In accordance with the regulations of Manual of Aviation Medicine in Taiwan, all pilots must complete and pass airframe-specific human centrifuge training courses before being assigned to the fighter wing. There are two different levels of training courses, the intermediate level and the advanced level, for pilots flying training jets and high-performance fighter pilots, respectively [18].

After strict administrative examination, the authority approved this study to be conducted by using centrifuge training data. The data sources in this study were the training worksheet and video recordings of each trainee during centrifuge training. Information was extracted from the above databases from 1 January 2011, to 31 December 2019. After strict anonymization and encryption procedures, each trainee was assigned a nonspecific number, and no information can be linked to individual trainees outside the databases to protect their privacy.

### 2.2. Study Participants

Participants in the database with the identity of “Air Force Academy male student pilots” who were undergoing “intermediate high G training” were selected. The intermediate high G training includes a lecture section and centrifuge training section. Approximately two weeks before centrifuge training, a four-hour classroom lecture on the physiological effects of acceleration forces, characteristics of and countermeasures against GLOC, factors related to diminished G tolerance, and correct AGSM technique was provided. After the lecture, APRL aviation physiologists demonstrate the proper AGSM to student pilots and then instruct student pilots to practice the AGSM.

This study was approved and classified into a low-risk group by the ethics committee of the Institutional Review Board of Kaohsiung Armed Forces General Hospital in Kaohsiung City, Taiwan (No. KAFGH 108–018). Because all data of this study were de-identified and anonymized, the protocol was determined to be exempt from informed consent.

### 2.3. Training Protocol

The human centrifuge was used to simulate a high G environment on the ground and was manufactured by the French Latécoère company in 1994. The length of arm was 25-feet long; the maximum G value and the onset rate were 15G and 6G per second, respectively. The seatback angle configuration is adjustable based on the aircraft type and training course of each trainee.

For centrifuge training, trainees must complete the following profiles in one training day: (a) a gradual onset run (onset rate: 0.1 G/second) to test the subject’s relaxed G tolerance (RGT) and straining G tolerance (SGT); (2) a rapid onset run (onset rate: 3 G/second) to 6G for 30 s to practice the correct AGSM; (3) a rapid onset run (onset rate: 3 G/second) to 7.5G for 15 s to ensure the trainee’s ability to effectively execute the AGSM and tolerate a high G environment; and (4) a rapid onset run (onset rate: 3 G/second) to 6G for 10 s to check six positions to accustom the pilot to performing the AGSM in abnormal postures [19]. During the training, the five-bladder anti-G suit is inflated for profiles 2 to 4. Between the profiles, participants had a two-minute rest and recovery at a 1.4G idle run.

The highest G level and target goal of the intermediate high G training is 7.5G for 15 s (7.5G profile). Thus, we explored the outcome of the 7.5G profile and the associations with the collected variables in this study. Exclusion criteria included no 7.5G profile attempt due to physiological discomfort (nausea, vomiting, pain, etc.) during the training (*n* = 7), incomplete individual data (*n* = 2), and the loss of HR signal on the video recordings (*n* = 19). In total, data from 873 participants were extracted for statistical analysis.

### 2.4. Definition of Outcome and Covariates

The training outcome of the 7.5G profile was classified into “fail” and “pass” categories. “Pass” meant that the subject could tolerate 15 s at the plateau of 7.5G without losing consciousness. “Fail” included conditions such as GLOC, near GLOC, or self-terminated G force before the completion of 15 s at 7.5G.

The anthropometric variables collected included age, height, weight, and body mass index (BMI) from the training worksheet. During the period of training, physiological data of trainees were monitored and recorded as electronic videos. The physiological parameters collected included HR, RGT, SGT, and AGSM score from the video recordings described as below:

HR: Real-time HR data were obtained from the twelve-lead electrocardiogram (Infinity CentralStation MS26800, Dräger, Telford, PA, USA), provided for the instructor’s reference and also stored as a file combined with the electronic video. We replayed the video to gain the different stages of HR defined as follows: resting HR, with recordings taken during five minutes of relaxation inside the cockpit as baseline values; HR during the 1.4G idle run, with recordings taken before the gradual onset run, 6G, and 7.5G profiles; and peak HR, with recordings taken during the 6G and 7.5G profiles. The oxygen reserve in the brain allows for the maintenance of consciousness for about 5 s during exposure to a high G environment [13,20]. Therefore, the HR increase percentage was computed as the peak HR during the first 1–5 s of 7.5G profile divided by the HR before the 7.5G profile.

RGT and SGT: Under the gradual onset run, RGT was defined as the G value when trainees detected the 100% loss of peripheral vision or the 50% loss of central vision by using the light bar in inside the gondola, at which point they started to perform the AGSM. SGT was defined as the G level after performing the AGSM, at which the trainees again met the criteria of vision loss mentioned above or the 9G upper limit [15]. We also reran electronic videos to record the values of trainees’ RGT and SGT.

AGSM score: A well-experienced aviation physiologist assessed the AGSM score during the 7.5G profile training by reviewing the electronic videos. The AGSM score consisted of rating the following four AGSM components: (1) taking a preparatory breath and holding it for 3 s; (2) forcefully exhaling and block against the glottis; (3) undertaking about 0.5 s of rapid air exchange; (4) performing equal and proper volume air exchange during the periods of inhalation and exhalation. The score range of each single component was from 1 to 4 points (1: very poor, 2: poor, 3: average, 4: good). A higher AGSM score indicates better AGSM operation (range from 4 to 16 points). 

### 2.5. Statistical Analysis

The physiological and anthropometric characteristics of the study participants are presented as the means ± standard deviations and percentages for all training information. Before comparison between the pass and fail groups, we examined whether data distributions met the assumptions of the statistical tests. Based on the test results of assumptions, chi-square or Fisher’s exact test were used for discrete variables and independent t test or Mann–Whitney U test were used for continuous variables. Variables with two-tailed *p* values <0.05 according to univariate tests were included in the final regression model.

The final model was constructed to address the study questions with multiple logistic regressions. The Hosmer and Lemeshow test was applied to determine the goodness of fit of the regression model. SPSS 24.0 software (IBM, Armonk, NY, USA) was used to manage the study data, and all tests were considered statistically significant at the *p* < 0.05 level.

## 3. Results

Within the 9-year period of data collection, there were 873 trainees made to withstand 7.5G for 15 s; among those, 44 trainees (5.04%) did not meet the pass criteria. As shown in Table 1, age and height were not different between the pass and fail groups. Compared with the fail group, the average weight and BMI were obviously higher in the pass group. The percentage of RGT under 4.5G and SGT under 6.5G in the pass group were both significantly lower than those in the fail group (21.71% vs. 63.64%; *p* < 0.001; 4.62% vs. 36.37%; *p* < 0.001). The proportions of AGSM scores below 8 points were 9.53% and 20.46% in the pass and fail groups, respectively.

Analysis of HR during centrifuge training showed that the HRs of the fail group at different stages were all significantly higher than those of the pass group except for the peak HR during the 7.5G profile (Table 2). The mean peak HR was 181.11 ± 21.84 beats per minute (bpm) in pass trainees and 172.48 ± 27.51 bpm in fail trainees. The HR increase percentage, defined as the peak HR during the first 1–5 s of the 7.5G profile divided by the HR prior to the 7.5G profile, was significantly smaller in failed trainees. The fail group also had a significantly higher percentage (fail group: 29.55%; pass group: 3.74%; *p* < 0.001) of trainees with a low HR increase (HR increase lower than 10%).

**Figure 1:**
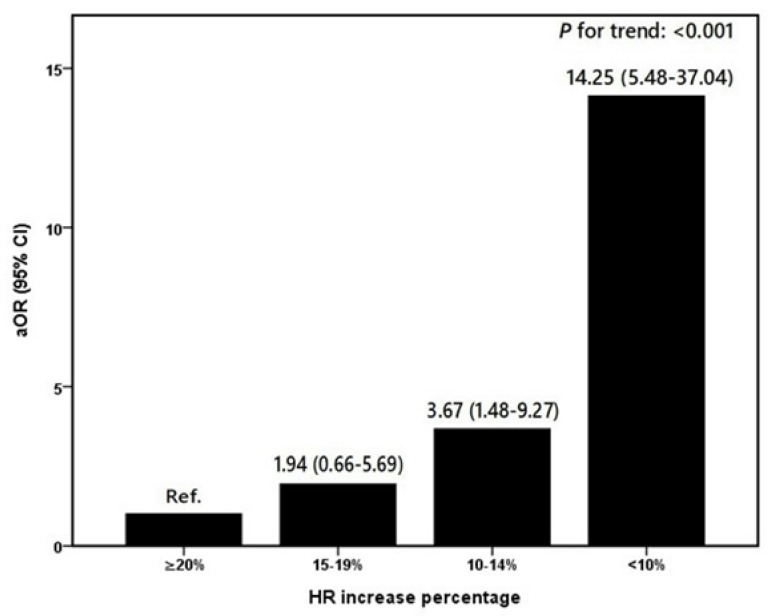
Dose-response effect of HR change on the failed outcome of the 7.5G profile. Model adjusted for age, BMI, RGT, SGT, and AGSM score. BMI: body mass index; RGT: relaxed G tolerance; SGT: straining G tolerance; AGSM: anti-G straining maneuver; aOR: adjusted odds ratio; CI: confidence interval. HR increase percentage: Peak HR during the first 1–5 s of the 7.5G profile divided by HR before the 7.5G profile.

As indicated in Table 3, the multiple logistic regression model showed that every unit increase in BMI decreased the chance of failure on the 7.5G profile by 21% (adjusted odds ratio (aOR): 0.79; 95% confidence interval (CI): 0.66–0.95). In terms of the physiological parameters, RGT was negatively correlated with failure of the 7.5G profile (<4.5G vs. ≥4.5G: aOR: 3.08; 95% CI: 1.51–6.32). SGT and the HR increase percentage were both independent factors strongly associated with the dependent variable. In particular, our results revealed that the possibility of failure during the 7.5G profile is dramatically increased by a smaller increase in HR (*p* for trend <0.001), as shown in Figure 1.

## 4. Discussion

Our work demonstrated that compared with the pass group, the fail group had a higher HR before reaching 7.5G and a lower peak HR while experiencing 7.5G. In other words, the proportion by which the HR increased during training was negatively and even dose-dependently associated with the outcome. The BMI, SGT, RGT, and AGSM score were significantly related to the dependent variable.

Previous studies have similarly shown that trainees’ HRs increase before centrifugation due to stress from anticipating the training [1]. We further found that the fail group had a higher HR at baseline and before the onset of the G load. This finding refuted the hypothesis based on the observation in the early stage of USAF centrifuge training that a lower resting HR might be a risk factor for GLOC [14]. There are several possible reasons to explain this disparity between our work and the USAF observation. First, the lower resting HR of trainees could be the result of an excessive amount of aerobic training that weakened the baroreflex activity. In the beginning of centrifuge training, the program of physical training was still not well-developed or emphasized for those pilots. Second, during the study period, student pilots in Taiwan were educated to undertake more anaerobic exercise and a moderate amount of aerobic training to improve cardiovascular fitness. Finally, psychological stress could also stimulate different HR responses between the pass and fail groups during the acceleration. Yun et al. indicated that trainees undergoing their first high G training experienced anxiety, and the success rate of 6G sustained for 30 s was negatively related to the depression level of the trainees [17]. In addition, we also observed that before the 7.5G profile, trainees in the fail group also had higher HR increases after the completion of 6G for 30 s profile (fail group: 137.61 ± 18.99 bpm; pass group: 146.11 ± 21.04 bpm; *p* = 0.004). This finding implies that the ability of the cardiovascular system to relax and recover could be one of the factors determining the G tolerance of jet pilots. From the literature review, trainees with faster recovery after the vigorous training would have optimal physical fitness and performance [21,22]. Therefore, fighter pilots should strengthen the relaxation and recovery ability from multiple G exposures by undertaking a well-designed physical training program.

From the perspective of exercise physiology, high G training is considered short-interval and high-intensity physical activity. The peak HR could be one of the physiological predictors of G tolerance during training. HR under sustained hypergravity reaches the maximum, depending on the G level, within seconds [13]. In agreement with the findings of previous reports, we also noted that in high G training, a maximum HR above 160 bpm was achieved during 7.5G exposure among all trainees [1]. Although GLOC trainees reached the target peak HR (>160 bpm), they failed to meet the training standard. The potential explanation is that in addition to HR, the fail group might have weaker cardiac performance than the pass group [23,24]. In future work, we will adopt a new study design to measure cardiac parameters such as stroke volume, cardiac output, ejection fraction, and contractility index to test the explanation mentioned above. Additionally, the peak HR among trainees in the fail group was still smaller than that in pass trainees. Low HR might be attributable to the delayed outflow of sympathetic tone, which is not able to overcome the reduced cardiac output induced by the G force before the depletion of the oxygen reserve [25]. We considered that the activation of the baroreflex is one of the important components in achieving high G tolerance during rapid onset run profiles. We decided to calculate the HR increase percentage by dividing the peak HR during the first 1–5 s of sustained 7.5G by the HR before the 7.5G profile. In a small-sample pilot study, we discovered the differences of hemodynamic changes between the GLOC group and the non-GLOC group during and before the 7.5G profile as measured by noninvasive impedance cardiography. Consistent with the former finding, we also revealed that the percentage of HR increase and the degree of HR acceleration were more obvious in the non-GLOC group after controlling for other covariates [26]. To further emphasize the association between the HR increase and the outcome variable in the stratified analysis, we discovered a significantly dose-effect relationship between them. Therefore, the increase or acceleration in HR might be adapted as an indicator of qualification during the rapid onset run in centrifuge training.

Previous studies have suggested that some physiological and anthropometric factors might be related to G tolerance, but there were controversial findings [15,16]. Data have shown that age has a positive effect on G tolerance in flight and during human centrifuge training [2,3,27]. However, our study population was composed of young student pilots of similar ages. As such, age should have minimal effects on their performance in centrifuge training. Past studies showed that taller pilots have reduced G tolerance because orthostatic stress is increased in taller individuals [15,16]. In the current study, results did not reveal a difference of height between the pass and fail groups in the univariate test. However, in line with several reports, greater weight seemed to have a positive influence on G tolerance [15,28]. Because we took the collinearity between the height and weight into consideration, we calculated BMI by using height and weight and assessed the effect of the BMI on G tolerance. Our result indicated that the BMI had a negative relationship with failure of the 7.5G profile training. This suggests a complex interaction of other physiological indices, for example, body composition, muscle, and fat mass, with height and weight on the G tolerance level [29,30,31]. The combined effect of those factors on G tolerance needs to be studied in future work.

In agreement with previous reports, we noted that RGT and SGT were both significantly related to the outcome of centrifuge training. SGT was examined as another strong negative predictor of GLOC in the centrifuge. AGSM remains one of the most important countermeasures against high G forces [32]. However, our multivariate model showed that the AGSM score had a borderline significant impact on G tolerance. There were several explanations for this discrepancy. First, the correctness of AGSM performed by trainees was evaluated by aviation physiologists before centrifugation. This policy could diminish the variation in AGSM among the trainees. Second, student pilots need to pass intermediate high G training before being assigned to a jet trainer. After the AGSM training course, trainees have the ability to correctly perform the AGSM. The AGSM technique is sufficient to enable them to tolerate the 7.5G challenge sustained for 15 s. Finally, AGSM techniques include a respiratory component and lower body muscle strain, but the AGSM score on the worksheet emphasizes the breathing component of AGSM. It is possible that the effect of leg muscle tensing on the training outcome has been overlooked.

There are some strengths and limitations of the current study. We conducted this first population-based study to review the training records of nearly 900 student pilots undergoing intermediate high G training. First, these results from such a large sample are more generalizable to young jet pilot populations compared with the results of other studies, shedding light on biological-specific factors affecting G tolerance. Second, compared with previous studies, this study explained the main effects of the independent variables on G tolerance more clearly by adjusting for potential confounders such as age, BMI, RGT, SGT, and AGSM scores. Third, we not only revealed that HR variation could be used as an indicator of the outcome but also clarified the dose-response relationship between HR and GLOC. Practically, our results emphasized that HR acceleration induced by the activation of the baroreflex also plays a crucial role in G tolerance during the rapid onset profile. We would like to compare our findings with the results of different markers that directly reflect cerebral perfusion under G stress [33,34,35]. However, good physical fitness has been shown to increase pilots’ AGSM efficiency and prolong the tolerated duration of simulated acrobatic combat maneuvers in the centrifuge [36]. However, there was no information on the trainees’ physical condition in our database. The influence of individual differences in physical fitness cannot be eliminated from this study. Finally, isometric contraction of the lower body muscles is the dominant component of the AGSM [20]. The performance of lower body muscles was not evaluated in this study due to the lack of data.

## 5. Conclusions

In summary, we analyzed a large database to explore the potential physiological response and anthropometric factors affecting G tolerance in the human centrifuge. Our findings suggested that the proportion of HR increase could be used as a parameter of the outcome of the 7.5G challenge and further quantified its dose-response effect on the dependent variable. In addition, BMI, RGT, SGT, and AGSM score are positively related to the tolerance for occupational hypergravity exposure.

## Figures and Tables

**Table 1:** Comparison of physiological and anthropometric characteristics between pass and fail groups.

Variables	Pass (*n* = 829)	Fail (*n* = 44)	*p* Value
Age (years)	23.53 ± 1.19	23.55 ± 0.66	0.920
Height (cm)	173.72 ± 5.32	174.11 ± 6.16	0.634
Weight (kg)	70.30 ± 8.58	66.09 ± 6.41	0.002
BMI (kg/m^2^)	23.27 ± 2.42	21.80 ± 1.78	<0.001
RGT (G)			<0.001
≥4.5	649 (78.29%)	16 (36.36%)	
<4.5	180 (21.71%)	28 (63.64%)	
SGT (G)			<0.001
≥6.5	794 (95.78%)	28 (63.63%)	
<6.5	35 (4.22%)	16 (36.37%)	
AGSM score			0.023
≥8	750 (90.47%)	35 (79.54%)	
<8	79 (9.53%)	9 (20.46%)	

BMI: body mass index; RGT: relaxed G tolerance; SGT: straining G tolerance; AGSM: anti-G straining maneuver; OR: odds ratio; CI: confidence interval.

**Table 2:** Univariate analysis of different stage heart rates (HRs) between pass and fail groups.

Variables	Pass (*n* = 829)	Fail (*n* = 44)	*p* Value
HR at baseline (bpm)	107.28 ± 17.37	116.09 ± 15.30	0.001
Peak HR during 6G profile (bpm)	168.45 ± 13.99	171.14 ± 11.95	0.211
HR before 7.5G profile (bpm)	137.61 ± 18.99	146.11 ± 21.04	0.004
Peak HR during 1–5 s of 7.5G profile (bpm)	181.11 ± 21.84	172.48 ± 27.51	0.012
HR increase percentage *			<0.001
≥10%	798 (96.26%)	31 (70.45%)	
<10%	31 (3.74%)	13 (29.55%)	

HR: heart rate; OR: odds ratio; CI: confidence interval. *: Peak HR during the first 1–5 s of 7.5G profile divided by HR before the 7.5G profile.

**Table 3:** Multivariate model of 7.5G profile outcome associated with independent parameters analyzed by logistic regression.

Variables	Pass (*n* = 829)	Fail (*n* = 44)	*β* ± SE	aOR (95%CI)	*p* Value
Age (year)	23.53 ± 1.19	23.55 ± 0.66	0.08 ± 0.18	1.08 (0.76–1.54)	0.661
BMI (kg/m^2^)	23.27 ± 2.43	21.80 ± 1.78	−0.23 ± 0.09	0.79 (0.66–0.95)	0.010
RGT (G)					
≥4.5	649 (78.3%)	16 (36.4%)		Ref.	
<4.5	180 (21.7%)	28 (63.6%)	1.13 ± 0.37	3.08 (1.51–6.32)	0.002
SGT (G)					
≥6.5	794 (95.78%)	28 (63.63%)		Ref.	
<6.5	35 (4.22%)	16 (36.37%)	1.93 ± 0.41	6.86 (3.08–15.25)	<0.001
AGSM score					
≥8	750 (90.47%)	35 (79.54%)		Ref.	
<8	79 (9.53%)	9 (20.46%)	0.73 ± 0.46	2.08 (0.84–5.13)	0.112
HR increase percentage *					
≥10%	798 (96.26%)	31 (70.45%)		Ref.	
<10%	31 (3.74%)	13 (29.55%)	2.29 ± 0.45	9.91 (4.11–23.88)	<0.001

Hosmer and Lemeshow test for goodness of fit: *χ^2^* = 12.65, *p* = 0.124; SE: standard error; BMI: body mass index; RGT: relaxed G tolerance; SGT: straining G tolerance; AGSM: anti-G straining maneuver; aOR: adjusted odds ratio; CI: confidence interval. *: Peak HR during the first 1–5 s of the 7.5G profile divided by HR before the 7.5G profile.
